# High-throughput near-infrared spectroscopy for detection of major components and quality grading of peas

**DOI:** 10.3389/fnut.2024.1505407

**Published:** 2024-12-09

**Authors:** Jingwen Zhu, Guozhi Ji, Bingyu Chen, Bangyu Yan, Feiyue Ren, Ning Li, Xuchun Zhu, Shan He, Zhishen Mu, Hongzhi Liu

**Affiliations:** ^1^Key Laboratory of Geriatric Nutrition and Health, Ministry of Education (Beijing Technology and Business University), Beijing, China; ^2^Global R&D Innovation Center, Inner Mongolia Mengniu Dairy (Group) Co. Ltd., Hohhot, Inner Mongolia, China; ^3^Inner Mongolia Enterprise Key Laboratory of Dairy Nutrition, Health & Safety of Inner Mongolia Enterprise, Hohhot, Inner Mongolia, China; ^4^Graduate School of Agriculture, Kyoto University, Kyoto, Japan

**Keywords:** pea, sensory quality, nutritional quality, quality grading, near-infrared spectral analysis, rapid test

## Abstract

Pea (*Pisum sativum* L.) is a nutrient-dense legume whose nutritional indicators influence its functional qualities. Traditional methods to identify these components and examine the relationships between their contents could be more laborious, hindering the quality assessment of the varieties of peas. This study conducted a statistical analysis of data about the sensory and physicochemical nutritional attributes of peas acquired using traditional techniques. Additionally, 90 sets of spectral data were obtained using a portable near-infrared spectrometer, which were then integrated with chemical values to create a near-infrared model for the basic ingredient content of peas. The correlation analysis revealed significant findings: pea starch displayed a substantial negative correlation with moisture, crude fiber, and crude protein, while showing a highly significant positive correlation with pea seed thickness. Furthermore, pea protein exhibited a significant positive correlation with crude fiber and crude fat. Cluster analysis classified all pea varieties into three distinct groups, successfully distinguishing those with elevated protein content, high starch content, and low-fat content. The combined contribution of PC1 and PC2 in the principal component analysis (PCA) was 51.2%. Partial least squares regression (PLSR) and other spectral preprocessing methods improved the predictive model, which performed well with an external dataset, with calibration coefficients of 0.89–0.99 and prediction coefficients of 0.71–0.88. This method enables growers and processors to efficiently analyze the composition of peas and evaluate crop quality, thereby enhancing food industry development.

## 1 Introduction

The pea (*Pisum sativum* L.) ranks as the fourth-largest legume crop globally, cultivated in more than 90 countries. In China, it is produced across 20 provinces and regions, including Sichuan, Yunnan, Henan, and Gansu (1–4). Renowned for their high yield and cost-effectiveness, peas are a primary source of commercial protein, offering a plethora of nutrients such as protein (20–25%), starch (36.9–49%), dietary fiber (14–26%), non-starch polysaccharides (12–24%), and lipids (1.2–2.4%), alongside significant levels of minerals like potassium, magnesium, and calcium (2.3–3.4%) (5). Pea seeds and pods contain essential bioactive compounds, including polyphenols, primarily flavonoids and phenolic acids, which are predominantly concentrated in the pea epidermis (6).

Conducting a thorough investigation into the relationship between the physicochemical and nutritional properties of pea raw materials is crucial for aiding food companies in the development of products with targeted functionalities. A significant number of researchers have conducted these investigations. For instance, there exists a negative correlation between the content of straight-chain starch in peas and the *in vitro* digestibility of pea protein. In contrast, the content of slowly digestible starch positively correlates with this digestibility (7). Lipid content similarly influences the functional properties of pea protein concentrates and isolates (8).

The traditional approach to identifying fundamental components in peas is characterized by its complexity, high cost, and protracted duration, which may introduce health and environmental hazards associated with the use of chemical reagents. Traditional approaches for assessing physicochemical and nutritional quality indicators in peas, while authoritative and comprehensive, are complex, time-consuming, inflexible, and heavily reliant on equipment. This may hinder the development of new products with specific functions (9). Although benchtop near-infrared (NIR) spectroscopy has been used for analyzing pea protein, it suffers from slow detection speeds and complicated data processing (10). Other scholars have used chromatographic techniques to detect physicochemical nutrients in peas, but chromatographic techniques have the disadvantages of relatively weak qualitative ability, complex operation, high cost, and great influence by the environment (11, 12). There is an urgent requirement for a fast, portable, and flexible instrument that provides real-time feedback, ensures sample protection, and promotes sustainable use. The device should facilitate simple operation and easy maintenance while ensuring accurate data recording to minimize human error. This instrument aims to detect the fundamental components in peas and investigate the correlation between pea qualities, enabling the rapid classification of pea varieties and the identification of those suitable for processing.

The NIR spectroscopy, which capitalizes on the molecular vibrations of asymmetric molecules like CH_3_-CH_2_-OH or H_2_O, is exceptionally suited for analyzing organic compounds in plant biomass that are NIR active, including bonds like O-H, C-H, C-O, C-O-H, N-H, and C=C (11–13). Not only that, but Near-infrared spectroscopy detection technology makes up for the shortcomings of traditional detection technology, such as slow detection speed, complex sample pre-treatment steps, which destroy the integrity of the sample, and the detection process may require the use of a large number of chemical reagents, which can cause environmental pollution and other shortcomings. Traditional detection technology typically focuses on specific analytical indicators, necessitating multiple tests to mitigate the risk of chance results. In contrast, near-infrared spectroscopy enables the simultaneous determination of multiple components within a sample, making it suitable for rapid quality control across various sectors, including food, pharmaceuticals, the chemical industry, and agriculture (14–16). At the same time, this study used the portable NIR tachymeters as an optimal solution for rapid and low-cost analysis, offering the development and validation of calibration models that rival conventional and benchtop NIR tachymeters in accuracy (17).

This study developed a rapid and straightforward method utilizing near-infrared spectroscopy to correlate the organoleptic, nutritional, and processing qualities of peas, facilitating the selection of specialized pea varieties. This will aid breeding specialists in developing specialized varieties and support processors in establishing a specialized ingredient base, thereby enhancing the utility and application of peas in the food industry.

## 2 Materials and methods

### 2.1 Sample collection and preparation

In this study, we selected naturally air-dried, high-quality pea seeds, including different seed shapes and seed colors, from the northern (e.g., Hebei, Shandong), southwestern (e.g., Sichuan), southern (e.g., Yunnan), and northwestern (e.g., Ningxia) provinces of China. The extensive geographic distribution of these provinces, coupled with their diverse climates, soils, and ecological environments, may result in variations in growth characteristics and adaptations among pea varieties in each region. Utilizing geographically differentiated pea seeds for modeling enhances the accuracy of study results. Secondly, these regions play a crucial role in pea cultivation. Selecting these primary production areas as research samples ensures the representativeness and applicability of the research findings, facilitating their implementation in broader agricultural production practices. More photographs of the pea samples are shown in Supplementary Figure S1. The experimental pea samples were later removed from impurities and broken grains and stored at 4°C (Qingdao Haier Biotechnology Co., Shandong, China). Before scanning the spectra, the test pea samples were placed in the same environment as the portable NIR tachymeter for 24 h. The aim was to align the sample environment with the instrument operating environment (18). Near-infrared reflectance spectra of peas were collected and the content of their basic components was determined sequentially. Correlation analyses of sensory quality, physicochemical, and nutritional quality of different varieties of pea seeds were conducted. A sketch of the workflow for this study is shown in Supplementary Figure S2.

### 2.2 Sensory quality analysis of single pea seeds

Pea seed size (length, width, and thickness), 100-seed weight, color, seed shape, and other characteristics have a significant impact on seed quality. The dimensions of pea seeds (length, width, and thickness) and the 100-seed weight are directly correlated with the nutritional reserves of the seeds, serving as critical indicators for evaluating their fullness and nutritional quality (19–21). Pea seeds exhibit greater nutritional value when they are of moderate size and possess an optimal 100-seed weight (22). At the same time, the color of high-quality pea seeds should be yellow-green or gray-green, and the shape of the seeds should be complete, full, with no cracks, and the epidermis should be glossy, not sticky (23). Pea seeds exhibiting these characteristics signify maturity and enhanced nutritional value (22). In the quality grading process, seeds that are of moderate size, possess a 100-seed weight, exhibit standard color, and have a typical seed shape are generally classified as high-quality seeds due to their superior growth stability (22).

In this study, the width, length, and height of different varieties of pea seeds were measured using vernier calipers (Shanghai Tool Works Co., Ltd., Shanghai, China), and the measurements were repeated five times and the mean values were recorded (24). The 100-seed weights of different pea varieties were weighed by an analytical balance (Osho International Trading, Shanghai, China), repeated three times, and averaged (25). The color and seed shape of different pea varieties were classified according to previously reported results (26).

### 2.3 Compositional analysis

The basic compositional content of peas was determined according to conventional methods: starch, crude protein, moisture, crude fiber, and crude fat. Each chemical analysis was repeated three times, and the results were averaged for data analysis. Starch was determined using a fully automatic starch tester (FOSS, Hillerød, Denmark) (27). Crude protein was determined by the Kjeldahl method (28) with a conversion factor of 5.46 on a 2,300 nitrogen analyzer (FOSS, Hillerød, Denmark). Moisture was tested by the drying method (29). Crude fat was determined by an automatic cable-type total fat analyzer (FOSS, Hillerød, Denmark) (30). Crude fiber was determined by a semi-automatic fiber analyzer (FOSS, Hillerød, Denmark) (31).

### 2.4 Portable NIRS and spectral collection

Portable near-infrared velocimeter using a 10 W halogen lamp source in the spectral range 908 nm to 1,676 nm with a sampling interval of 6 nm, based on the Micro-NIR spectrometer (Manufacturer: VIAVI Solutions Inc.) (32). Detailed parameters of the portable NIR tachymeter are shown in Supplementary Table S1. The device is controlled by a tablet computer (Surface, Microsoft Corporation, United States) and collects spectral data using a linear variable filter (LVF) in transmission mode. This high-throughput, non-destructive testing device includes components such as a case, cover, display, spectrometer, and a Teflon reference whiteboard. Before spectral acquisition, system parameters were set up using Micro-NIR Pro 2.4 software (VIAVI Solutions, United States). Different chemical components have specific absorption peaks in the near-infrared (NIR) band, so choosing an instrument that can fully cover the wavelength range of these peaks is critical to improving detection accuracy (33). The wavelength range of the spectra collected by the portable near-infrared spectrometer used in this experiment covers exactly the full range of absorption peaks of peas (34). In addition, an appropriate extension of the integration time can reduce the impact of noise on the spectral data, thereby improving the reliability of detection (33). Therefore, in this study, the spectral integration time of the spectrometer was set to 12.7 ms, which is higher than that of a common benchtop near-infrared spectrometer, to obtain all the spectral data of the pea sample in the range of 908–1,676 nm (35). During spectral acquisition, room temperature must be maintained at 25°C, and the temperature of the spectrometer must be consistent when collecting spectra for each sample to prevent baseline drift and ensure accurate spectral information (36). Therefore, when using the portable NIR tachymeter, first turn on the machine and preheat it at room temperature for 1 h. The spectrometer temperature will reach a constant before measurement. Experimental pea samples were placed in the sample cup (50 mm high; 51 mm in diameter), and gently shaken to distribute the seeds uniformly, and each pea sample was scanned five times. The sample cup rotated at a certain angle for each scan, and the process was repeated three times to average the result. Due to sample heterogeneity and distribution heterogeneity, the spectra of different sample parts are different, so it is necessary to mix several times and scan several times to average the spectra as the original spectra to reduce the error.

### 2.5 Construction of model

The models were constructed and analyzed using the Unscrambler X 10.3 software (CAMO, Norway) and MATLAB R2021a software (MathWorks, United States).

#### 2.5.1 Removal of outliers

The Mahalanobis distance is a kind of generalized squared distance, which is based on the theory of multivariate normal distribution and effectively takes into account the three parameters of mean, variance, and covariance, and it is a comprehensive indicator that can comprehensively describe the overall multivariate structure (32). Principal component analysis (PCA) is a method used in mathematics for dimensionality reduction, where a set of multiple variables are recombined into a new set of mutually unrelated composite variables by orthogonal transformation to reduce the number of variables, and the information of the original variables is represented by selecting the principal components (PCs) whose contribution accounts for the larger cumulative contribution (37). The combination of PCA with Mahalanobis distance, whereby principal component scores are used in place of the original data to calculate Mahalanobis distance, not only reflects all the data information but also compresses the number of variables participating in the calculation of Mahalanobis distance and ensures that there is no covariance in the M-matrix (38).

This study used PCA combined with Martensitic Distance for outlier rejection work on all pea spectral data before spectral pre-processing. MATLAB R2021a software was used for the procedure writing of this method.

#### 2.5.2 Spectra pretreatment

When calculating the results using NIR spectroscopy, factors such as the particle size of the sample to be measured, the homogeneity of the internal structure of the sample, the stability of the sample itself, and the noise of the instrument itself during the detection of the sample will all have an impact on the results (39, 40). To reduce the influence of the intrinsic factors of the instrument and the sample itself on the accuracy and stability of the quantitative analysis model, improved the quality of acquired spectral data, it is necessary to use a highly selective method to pre-process the NIR spectral data first, and then build the corresponding quantitative analysis model on this basis.

Commonly used spectral preprocessing methods mainly include Smoothing, Normalization, Baseline, standard normal variable (SNV), Detrend, multiple scattering corrections (MSC), 1st Derivative, and 2nd Derivative (41). The derivative algorithm is used to eliminate the effect of the background or drift of the measuring instrument on the signal. The MSC and SNV transform belong to the scattering correction, which is used to eliminate the effect of scattering on the spectrum caused by the uneven distribution of particles and differences in particle size. Smoothing is mainly used to remove random noise from the spectral signal (33, 42, 43). In this study, two, three, or even four of the above seven single preprocessing methods were selected to combine for composite spectral preprocessing, and the best spectral preprocessing method was finally selected.

#### 2.5.3 Model building and evaluation

Near-infrared spectroscopy is an indirect analysis technique, through the establishment of a calibration model to quantitatively or qualitatively analyze unknown samples, the main process includes the establishment of the model and the detection of unknown samples. The specific NIR spectroscopy techniques analyzed are shown in Supplementary Figure S3 (44, 45). This flowchart was drawn by Figdraw^1^.

Quantitative analysis aims to establish stable and reliable models for quantitative analysis. The modeling consists mainly of the choice of calibration methods and the determination of chemical values, the choice of stoichiometry, and the prediction of unknown samples. Among them, the selection of calibration methods is the core of chemometrics. Currently, the commonly used calibration methods are principal component regression (PCR), partial least squares (PLS), multiple linear regression (MLR), artificial neural network (ANN), and topology, TP, etc. (46). Partial least squares regression (PLSR) is a regression modeling method that addresses the interdependence between two sets of multi correlated variables. It is used to investigate the use of one set of variables (independent or predictor variables) to predict another set of variables (dependent or response variables) (3). PLSR is an appropriate methodology for modeling when the number of variables in the two groups is considerable and linearly correlated, and the number of observations is limited. Compared with traditional multiple linear regression, PLSR can effectively solve the problem of multicollinearity between variables, has the advantages of simple calculation, high prediction accuracy, and easy qualitative interpretation, and its constructed model can more accurately identify the effective information (3, 47). This technique has been successfully used to predict the functional properties of grains, such as the content of protein subunits, gelatinization, solubility, etc. (32). Therefore, in this study, the preprocessed spectral and chemical values were analyzed by PLSR.

In this experiment, the Kennard-stone algorithm (3:1) is used to divide the correction and validation set samples during the modeling process (13, 48): the Kennard-stone algorithm is one of the commonly used techniques for selecting the samples of the correction set and the samples of the validation set, which means that all the samples are considered to be the candidate samples of the correction set, and the first one to be selected is the one with the furthest Euclidean distance from a pair of samples is chosen first, then the two samples that are farthest as well as closest to the selected samples are found by calculating the Euclidean distance from each of the remaining samples to each of the known samples in the validation set, and these two samples are then selected into the correction set, and the process is repeated until the desired number of samples is reached. The remaining samples are used as the validation set to validate the model, and the procedure is repeated several times to obtain the correction sample correlation coefficient (*R*_c_^2^), root mean standard error of calibration (RMSEC), residual predictive deviation of calibration (RPDC), prediction sample correlation coefficient (*R*_p_^2^), root mean standard error of prediction (RMSEP), and residual predictive deviation of prediction (RPDP), which are the metrics for evaluating the performance of the model: *R*^2^, the larger residual predictive deviation (RPD), the smaller square error of calibration (SEC), the standard error of cross-validation (SECV), the square error of prediction (SEP) indicate better model performance (39, 46, 49).

## 3 Results and discussion

### 3.1 Results of sensory quality analysis of different pea varieties

**Figure 1:**
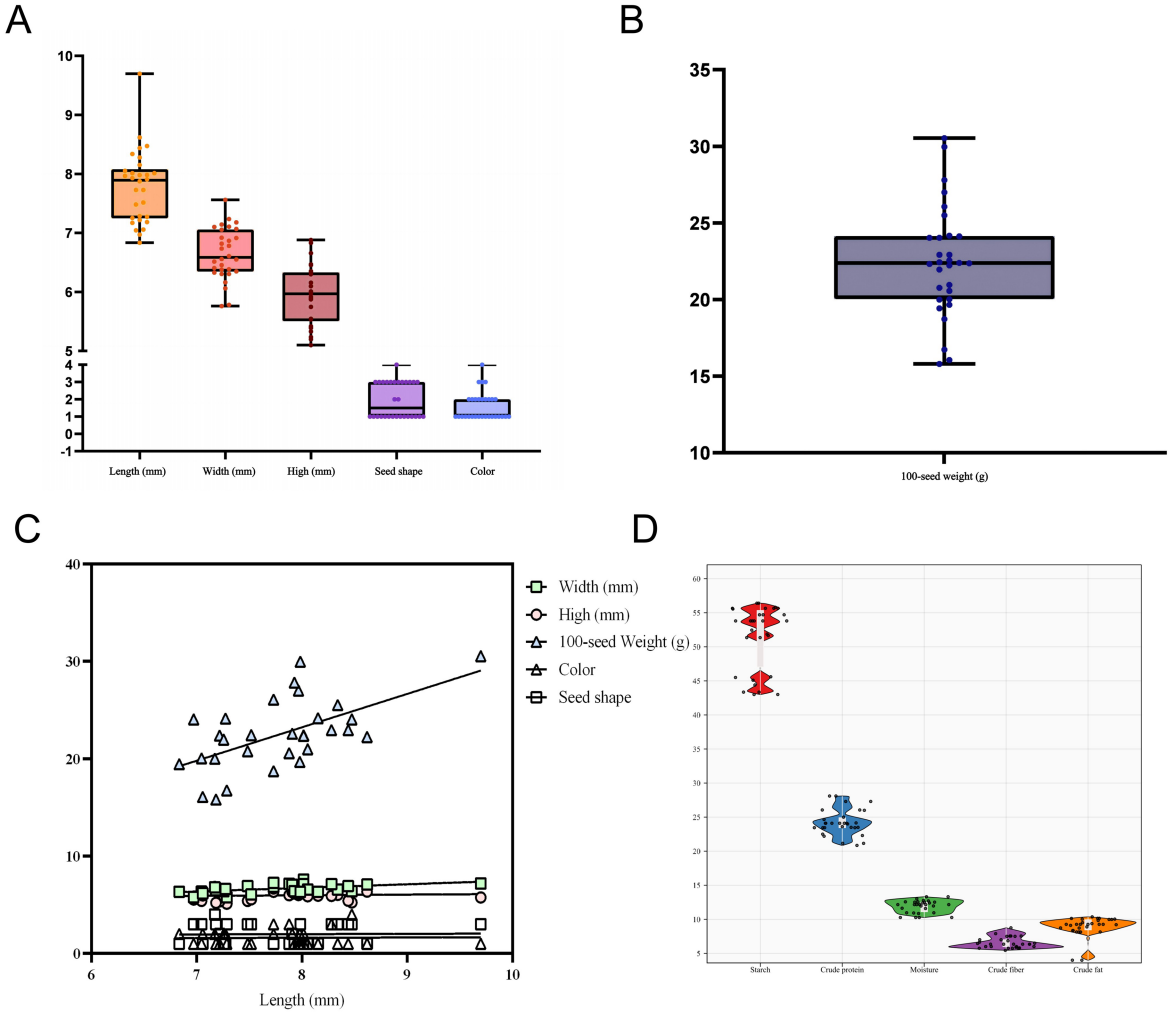
Box plots of pea sensory qualities (length, width, height, seed shape, color and 100-seed weight; the black line in the box is the mean) **(A,B)**; scatter plots illustrating the correlation between pea sensory qualities **(C)**; and distribution of basic pea constituents (starch, crude protein, moisture, crude fiber, and crude fat) **(D)**.

The distribution of organoleptic qualities (length, width, height, 100-seed weight, color, seed shape) of different varieties of pea seeds was shown by a box plot (Figures 1A,B). Pea seeds’ length varied from 6.83–9.69 mm in length (one outlier), 5.76–7.56 mm in width (no outlier), 5.10–6.88 mm in height (no outlier), and 15.80–30.53 g in 100-seed weight (one outlier) (Table 1). This is similar to the results of previous studies on the 100-seed weight as well as the length, width, and height of pea seeds (50, 51). Following an analysis of the morphology and odor of different varieties of pea seeds, it was found that the morphology was that of normal varieties without insect erosion, mold, impurities, or foreign matter, the shape of the seeds was mainly wrinkled, round, concave-rounded and flat-rounded, and the colors were mainly brown, green, flaxen and purple. The smell of pea seeds is the inherent flavor of peas, no smell. In summary, the morphology and smell of different varieties of pea seeds were normal, with no significant differences noted among the varieties. This is similar to the results of a previous study on seed coat color and seed shape of pea seeds (6).

**Table 1:** Descriptive examination of the chemical values of sensory quality and vital component composition of pea samples.

Factor	Scope of change	Average value	Coefficient of variation	Highest four points	Upper quartile	Lower quartile	Standard deviation
Seed shape	1.00–4.00	1.97 ± 0.19	0.53	1.00	1.50	3.00	1.03
Color	1.00–4.00	1.60 ± 0.15	0.51	1.00	1.00	2.00	0.81
100-seed weight (g)	15.80–30.53	22.47 ± 0.66	0.16	20.03	22.38	24.14	3.59
High (mm)	5.10–6.88	5.96 ± 0.09	0.08	5.51	5.97	6.33	0.49
Length (mm)	6.83–9.69	7.78 ± 0.11	0.08	7.24	7.89	8.07	0.61
Width (mm)	5.76–7.56	6.65 ± 0.08	0.07	6.35	6.59	7.06	0.44
Starch (g/100 g)	43.05–57.55	51.41 ± 0.83	0.09	45.58	51.40	54.70	4.56
Crude protein (g/100 g)	19.80–28.45	23.80 ± 0.38	0.09	22.28	23.48	25.03	2.10
Moisture (%)	10.14–13.31	11.94 ± 0.17	0.08	11.17	11.94	12.73	0.94
Crude fiber (g/100 g)	5.40–8.75	6.55 ± 0.16	0.13	5.79	6.55	6.89	0.85
Crude fat (g/100 g)	3.00–4.25	3.53 ± 0.58	0.09	3.30	3.53	3.78	0.32

**Figure 2:**
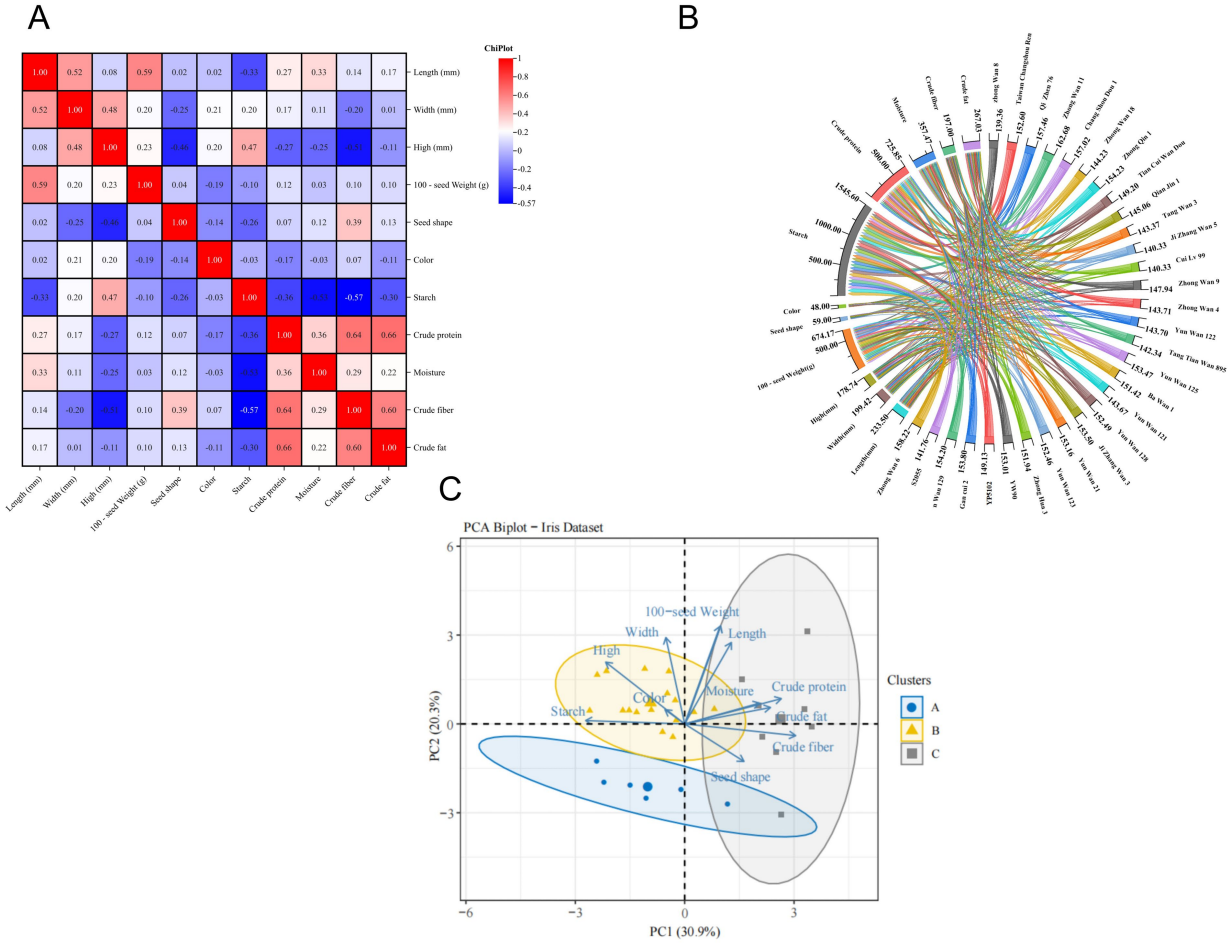
Heat map illustrating the correlation between pea sensory quality and fundamental ingredient content (darker blue color indicates weaker correlation, while darker red color indicates stronger correlation) **(A)**, chord plot **(B)**; PCA biplot of various pea samples (*n* = 30, distinct colored ovals represent different sample clusters, the length of the arrow indicates the rate of contribution of the indicator and the angle of the arrow indicates the correlation) **(C)**.

A scatter plot of pea sensory quality correlations was drawn using pea length as the horizontal coordinate. As shown in Figure 1C, pea width, high, 100-seed weight, color, and seed shape were positively correlated with pea length. At the same time, using the correlation analysis method as Pearson’s correlation analysis showed that the length of pea seeds was significantly and positively correlated with width (*r* = 0.52) and 100-seed weight (*r* = 0.59). This indicates that there is a close relationship between the morphology of pea seeds and 100-seed weight, and changes in the morphology of pea seeds will significantly affect the changes in 100-seed weight. The width and height of pea seeds were also significantly positively correlated (*r* = 0.48), indicating that the greater the width of pea seeds, the greater the height. However, the seed shape of pea seeds showed a significant negative correlation (*r* = −0.46) with height (Figure 2A).

### 3.2 Results of the content of basic components of different pea varieties

Conventional methods were used to measure the content of essential components in the pea samples used for modeling, and a distribution plot consisting of a box plot (black box in a violin) and a violin plot shows the distribution of the content of essential components in the peas (Figure 1D), with the corresponding chemical values shown in Table 1. The Violin diagram was mapped using ChiPlot^2^. Among the pea samples the variation of starch content ranged from 43.05 to 57.55% (no outlier), crude protein content ranged from 19.80 to 28.45% (no outlier), moisture content ranged from 10.14 to 13.31% (no outlier), crude fiber content ranged from 5.40 to 8.75% (no outlier), and crude fat content ranged from 3.00 to 4.25% (one outlier). These data are similar to the results of previous studies on the content of basic components of pea seeds (52). A comparison of current methods for testing pea nutritional quality indicators is shown in Table 2.

**Table 2:** Comparison of common technical methods for detecting physicochemical and nutritional quality indexes in peas.

Testing technology	Advantages	Disadvantages	Main testing methods	Test content	Conclusions	Bibliography
Chromatography	High sensitivity; high separation efficiency; high selectivity; rapid analysis; wide range of applications.	Relatively weak qualitative ability; the complexity of the operation; high cost; subject to environmental influences.	HPLC-MS/MS	Pea protein in meat products	The limits of detection (LODs) of the method were about 5 mg/kg meat product for pea protein.	(60)
			Hydroxyapatite chromatography-HPLC	Pea protein	The quantitative analysis of seed proteins from peas and many other seeds.	(61)
			UPLC-QTOF-MS and HPLC-QQQ-MS/MS	Phenolics in peas	The ethanol extracts of 10 peas mainly included 12 kinds of phenolic substances.	(11)
Spectroscopy	Simple; fast; cost-effective; no sample destruction.	Expensive; samples may require pre-treatment.	FT-MIR	Pea starch and pea carotenoids	The starch PLSR model correlated greater than 0.75, and carotenoids had a correlation of 0.71 for the validation sets.	(34)
			NIRS	Pea protein and methionine	The accuracy of prediction of methionine was ±0.01% and of protein ±0.76% of the whole peas with a commercially available near-infrared reflectance instrument. The time for testing each sample for both methionine and protein was 45 s.	(62)
				Physicochemical quality parameters of peas (color, firmness, total soluble solids, pH, total polyphenols, ascorbic acid and protein content)	The coefficients of determination in the external validation ranged from 0.50 to 0.88.	(63)
				Amino acid content in peas	The validation showed that 85–98% of the amino acid variance in the samples could be explained using NIRS.	(64)
			FT-IR and UV–Vis spectroscopy	Identifying green peas	The FTIR showed excellent performance (*r*_val_ > 0.93) in predicting adulterant levels with a standard error of prediction (SEP) of 0.66% for green peas. The UV–VIS predicted (*r*_val_ > 0.93) the adulterant levels with SEP 0.58% for green pea.	(65)
	High sensitivity; high specificity; fast and efficient; high sample recovery rate.	The test process is easily disturbed; difficult to operate.	SYBER Green qPCR	Pea allergens	The Pis s1 and Pis s2 are pea globulin storage proteins.	(66)
			TaqMan qPCR	Pea ingredients	Limiting of detection for the pea component up to 0.10% (mass fraction) of its content.	(67)
			The immunofluorescence method	Pea protein	The detection limit (LOD) of the method for pea flour was 0.50% addition, and for pea protein it was 0.001% addition.	(68)
			Hydride generation-atomic fluorescence spectrometry	Selenium content in peas	The detection limit was 0.10 ng/mL and the linear range was 0–80 ng/mL.	(69)
Conventional methods	Standardized; comprehensive; authoritative; widely applicable.	Complex; time-consuming; equipment-dependent; inflexible; lagging in updates.	Kjeldahl nitrogen determination (KND)	Pea protein	Pea protein (19.75–26.48%).	(11)
			Enzymatic hydrolysis	Pea starch	Pea starch (32.56–32.56%).	
			Powdered sulfuric acid	Pea dietary fiber	Pea dietary fiber (11.34–16.13%).	
			Soxhlet extraction method	Pea fat	Pea lipids (0.57–3.52%).	
Other methods	High specificity; high sensitivity; wide range of applications.	Higher cost; samples need to be handled; technology dependent.	A multiplex legume allergen detection assay (LADA)	Pea allergens and pea protein	Detecting intact units of allergenic proteins or at least larger fragments of allergenic proteins in food ingredients.	(70)

### 3.3 Combined analysis of sensory qualities and basic ingredient content

#### 3.3.1 Correlation analysis between sensory quality and basic ingredient content

The method of correlation analysis was taken as Pearson’s correlation analysis as represented in Figure 2A: the closer the graph is to blue, the weaker the correlation between the indicators; the closer the graph is to red, the stronger the correlation between the indicators. The results show that pea height had a highly significant positive correlation with pea starch (*r* = 0.47), but a highly significant negative correlation with crude fiber (*r* = −0.51); seed shape also showed a significant positive correlation with crude fiber (*r* = 0.39), which is similar to the results of previous studies (53). Additionally, there was a significant negative correlation between pea starch and moisture (*r* = −0.53), crude fiber (*r* = −0.57), crude protein (*r* = −0.36); there was a significant positive correlation between crude protein and crude fiber (*r* = 0.64), crude fat (*r* = 0.66). This may be because the higher content of crude fiber aids in the efflux of proteins and fats, thereby increasing the protein extraction rate. Crude fiber was highly significantly and positively correlated with crude fat (*r* = 0.60). In summary, the selection of low-fat, high-protein pea varieties should consider height varieties, a trend also observed in sweet potato leaves (54). The heat map was mapped using ChiPlot (see Footnote 2).

According to the chord diagram (Figure 2B), it can be seen that there is a significant relationship between pea sensory quality and physicochemical and nutritional quality and that one indicator may constrain or contribute to changes in several indicators, so that trends in one indicator may indicate trends in several indicators the contents of basic components of different varieties of peas are correlated, with the largest pathways for starch and crude protein. At the same time, it also demonstrates that peas have a higher starch content and protein content.

#### 3.3.2 Principal component analysis and cluster analysis of sensory quality and essential component content

The PCA was used to explore the relationship between the content of essential components in different pea samples. Figure 2C shows the PCA labeled plot (PCA scoring labeled plot combined with K-means clustering as an unsupervised clustering method). The sum of PC1 and PC2 was 51.2%. At the same time, As can be seen in the Figure 2C the pea starch was negatively correlated with moisture, crude fiber, crude protein, and crude fat because the angles of their arrows were all greater than 90°. This is similar to previous studies (36, 40–42). The study also found that crude protein was also negatively correlated with the thickness of pea seeds. The angles of crude protein, crude fat, and moisture are all less than 90°, indicating a positive correlation between these three indicators, which is consistent with previous findings (52, 55). In summary, pea varieties with higher crude protein content tend to be lower in starch. In addition, the arrows pointing to PC2 have the longest arrow lengths for pea height and starch, indicating that pea height and starch contribute the most to PC2.

K-means as an unsupervised method was used to cluster the peas based on all components analyzed. As shown in Figure 2C, the 30 pea varieties were categorized into three classes. Starch, protein, and fat are categorized into three separate classes. The blue dots represent the first category, which consists of six varieties from Hebei, Ningxia, Yunnan, and Sichuan, respectively. Compared with other experimental pea varieties, it is characterized by lower fat content, which can be classified into three levels according to the fat content in total, ≤5% for the first level, 5–9% for the second level, and ≥9% for the third level, and therefore it is recommended to use “*Cui Lu 99*” to make a low-fat functional food (56). The yellow triangle represents the second category, including 16 varieties from Hebei, Yunnan, Sichuan, and Shandong, which are characterized by higher starch content and larger seed shape and can be classified into three levels according to the starch content, with the content ≥55% for the first level, 45–55% for the second level, and ≤45% for the third level. It is therefore recommended that “*Ba Wan 1*” be used for canned goods, dried fruit, and other products, or processed into pea flour for bread, biscuits, and other products (56). Gray squares represent the third category, consisting of eight varieties from Shandong and Ningxia, which are characterized by high crude protein content and can be divided into three levels according to protein content, with ≥25% as the first level, 20–25% as the second level, and ≤20% as the third level. For this reason, it is recommended to use “*Qi Zhen 76*” as a pea protein powder or pea protein meat substitute to provide a source of protein for dieters or vegetarians (56). Specific pea variety screening can be seen in Table 3.

**Table 3:** Classification and grading status of peas (*n* = 30).

Classification of varieties	The first type			The second type			The third type		
Norm	Fat content			Starch content			Protein content		
Classification of indicators	The First level: ≤5%	The second level: 5–9%	The third level: ≥9%	The First level: ≥55%	The second level: 45–55%	The third level: ≤45%	The First level: ≥25%	The second level: 20–25%	The third level: ≤20%
Foods suitable for preparation	Low-Fat Functional Foods			Used in canned goods, dried fruit, and other products, or processed into pea flour for bread, biscuits, and other products			Pea protein powder or pea protein meat substitute		
Variety name	*“S2055,” “Tian Cui Wan Dou,” “Cui Lu 99,” “Yun Wan 122,” “Tang Tian Wan 895,” “Yun Wan 121”*			*“Zhong Qin 1,” “Qian Jin 1,” “Tang Wan 3,” “Ji Zhang Wan 5,” “Yun Wan 125,” “Ba Wan 1,” “Yun Wan 128,” “Ji Zhang Wan 3,” “Yun Wan 21,” “Yun Wan 123,” “Zhong Hua 3,” “YW90,” “YP5102,” “Gan Cui 2,” “Yun Wan 129,” “Zhong Wan 6”*			*“Zhong Wan 8,” “Tai Wan Chang Shou Ren,” “Qi Zhen 76,” “Zhong Wan 11,” “Chang Shou Dou 1,” “Zhong Wan 18,” “Zhong Wan 9,” “Zhong Wan 4”*		

### 3.4 Analysis of near-infrared spectral data

**Figure 3:**
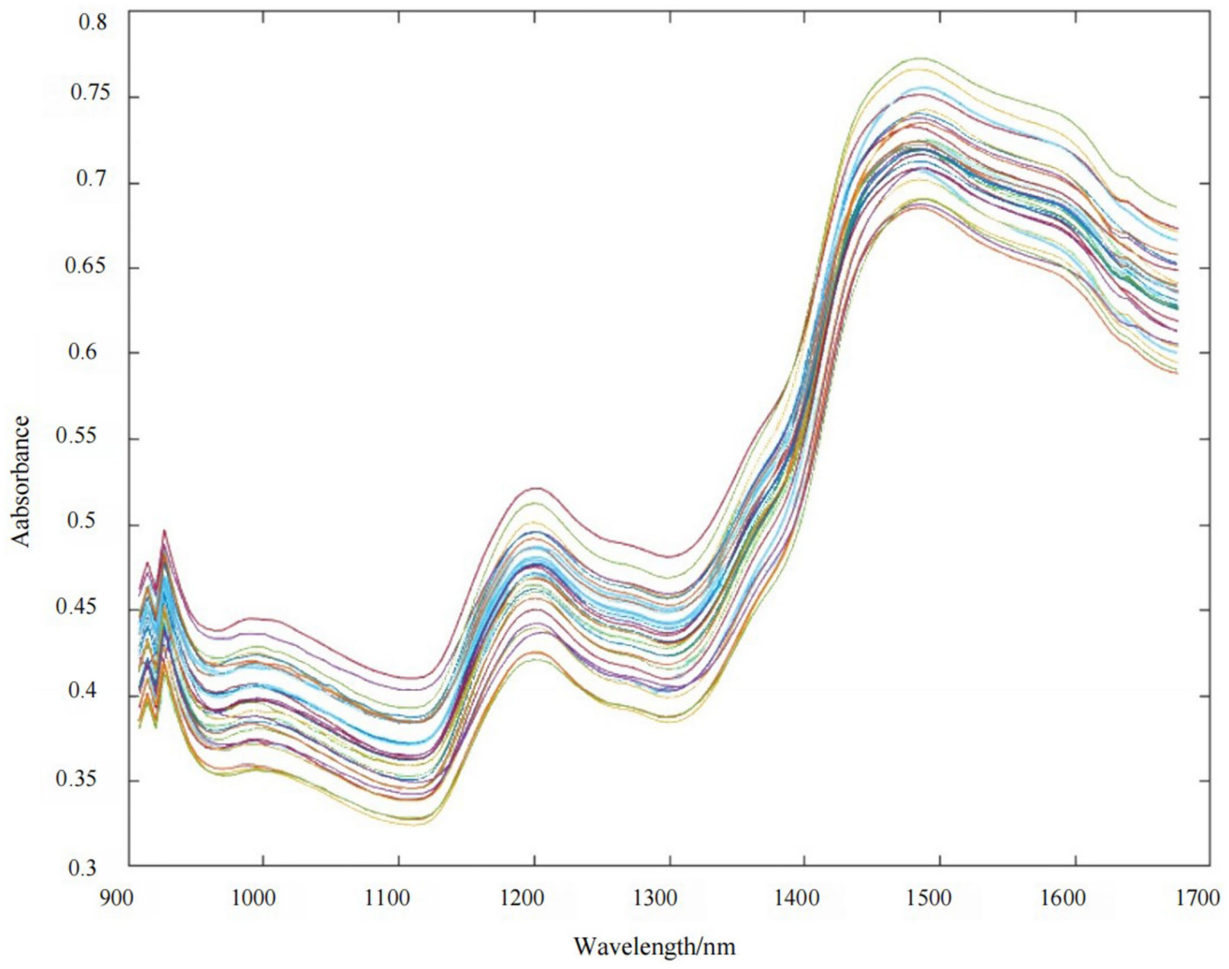
The near-infrared spectrum of the pea sample set (*n* = 90).

**Figure 4:**
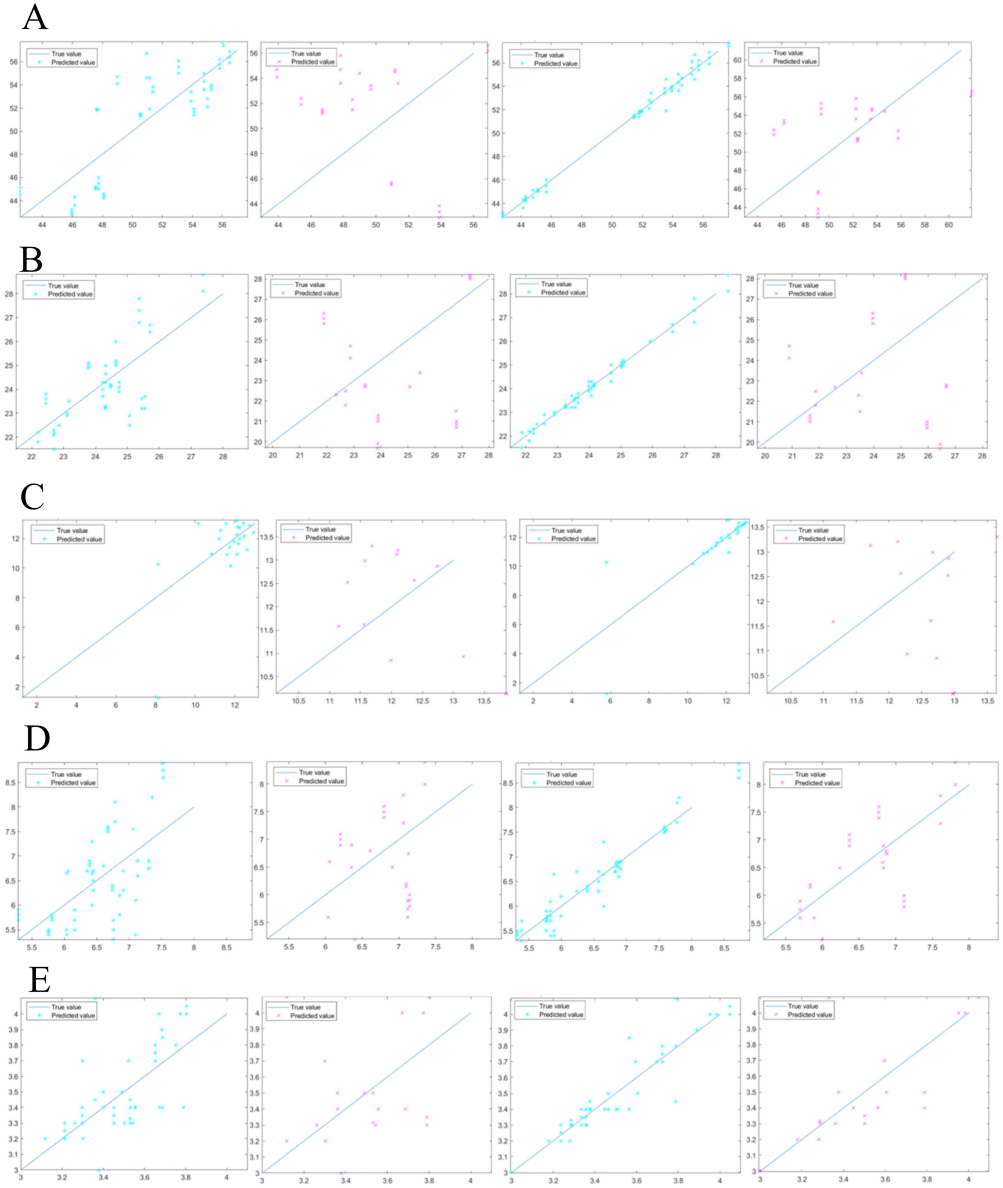
Comparison of untreated and pretreated models for pea starch **(A)**; comparison of untreated and pretreated models for pea crude protein **(B)**; comparison of untreated and pretreated models for pea moisture **(C)**; comparison of untreated and pretreated models for pea crude fiber **(D)**; comparison of untreated and pretreated models for pea crude fat **(E)**.

The raw NIR spectra of the pea samples are shown in Figure 3, where the horizontal coordinates are the wavelengths (908–1,676 nm) and the vertical coordinates are the absorbance. The analysis of spectral data plots indicates that the near-infrared diffuse reflectance spectra of all pea samples exhibit similar trends, primarily characterized by the doubling and combining frequency information of C-H, O-H, and other chemical bond stretching interactions. The presence of numerous hydrogen-containing groups, including C-H, O-H, and N-H, in the raw material of peas accounts for their near-infrared absorption properties. Specifically, the 927 nm comes from the C-H tertiary telescoping multiplication of methylene (57), while the 952 nm is mainly from the O-H secondary telescoping multiplication (58). The 1,125 nm and 1,181 nm come from the C-H secondary telescoping multiplication, respectively (59) and C-H secondary stretching multiples in HC=CH. The literature also reports that the C-H secondary stretching multiplier in CH_2_-CH_2_ produces a 1,212 nm absorption, while 1,243 nm comes from a 3 × C-H stretching combinatorial frequency (57). The 1,280 nm absorption is produced by the multiplication frequency of O-H, while the absorption of the combinatorial frequency of C-H_3_ is produced at 1385 nm (57). The 1,428 nm absorption is generated by the N-H primary stretching frequency doubling, etc. (57). The PLSR model for the raw spectra of the basic ingredient content of peas was established based on the full wavelength (908–1,676 nm), as shown in Table 4 and Figure 4, and the *R*_c_^2^ of the PLSR model was 0.34–0.68, the RMSEC was 0.22–2.62, the *R*_p_^2^ was 0.00–0.21, and the RMSEP was 0.37–6.65, which indicated that the raw spectra of peas had a PLSR model had poor predictive performance. Therefore, either a single preprocessing method or a composite preprocessing method is needed to analyze the raw spectral data. Thus, the spectrally optimal preprocessing method was selected, leading to a more accurate and stable PLSR prediction model for pea basic ingredient content.

**Table 4:** Construction and validation of PLSR models for raw and optimally preprocessed spectra.

Index	Raw/pretreatment method	Correction set (*n* = 54)			Verification set (*n* = 26)		
		*R*_C_^2^	RMSEC	RPDC	*R*_P_^2^	RMSEP	RPDP
Starch	Raw/Derivative (2nd)	0.68/0.99	2.62/0.47	1.78/3.87	0.09/0.77	6.65/0.86	0.65/1.70
Crude protein	Raw/Derivative (2nd)	0.53/0.98	1.12/0.21	1.46/3.82	0.00/0.77	3.33/0.24	0.81/1.82
Moisture	Raw/Derivative (2nd)	0.34/0.85	1.35/0.90	1.23/1.90	0.21/0.71	1.52/1.00	0.66/1.18
Crude fiber	Raw/Derivative (1st) + Derivative (2nd)	0.38/0.93	0.71/0.25	1.27/3.70	0.01/0.72	0.88/0.59	0.93/1.19
Crude fat	Raw/Derivative (1st) + Derivative (2nd) + Detrend	0.39/0.89	0.22/0.09	1.29/1.93	0.03/0.88	0.37/0.25	0.81/1.92

### 3.5 Analysis of basic components in pea

**Figure 5:**
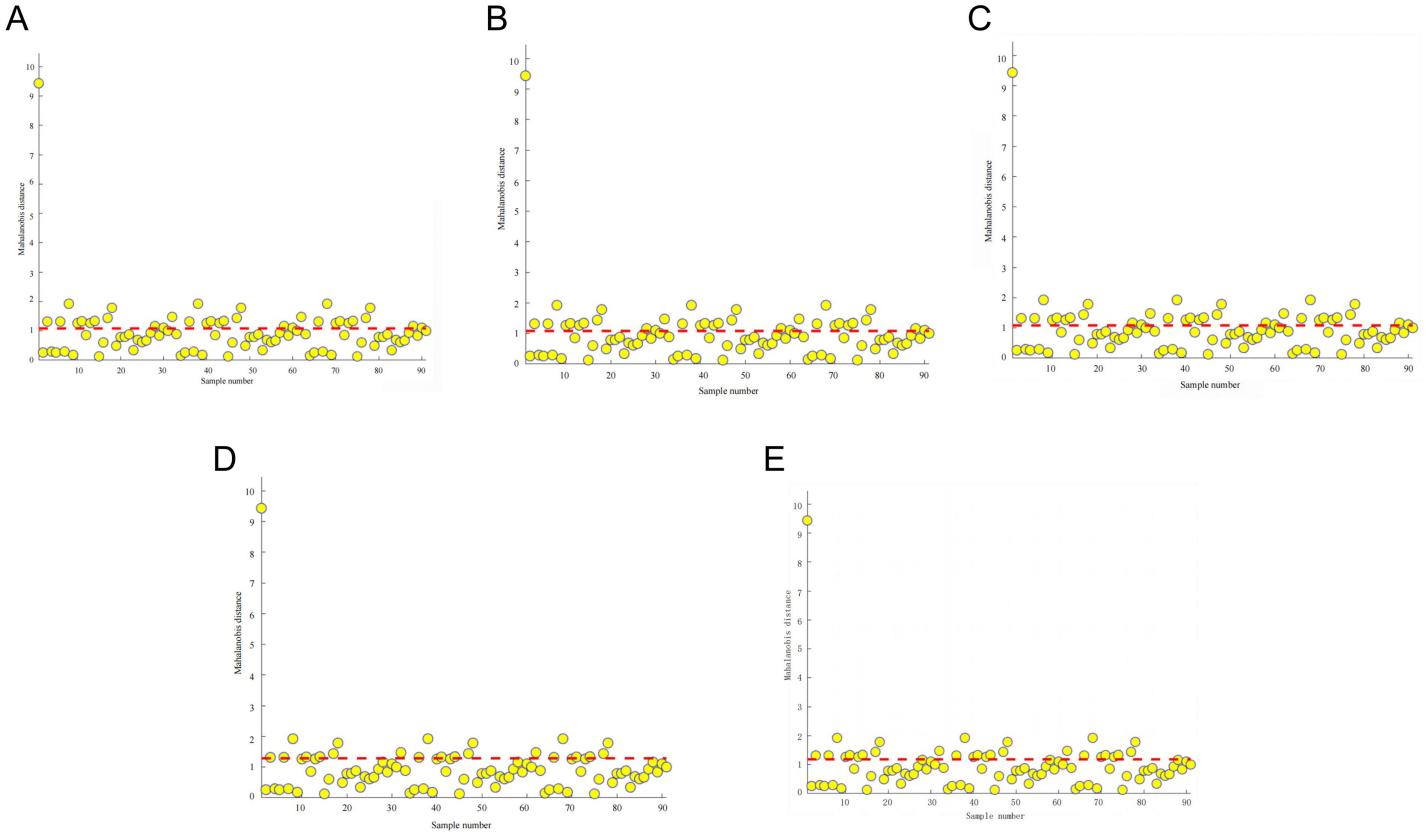
Mahalanobis distance and appropriate thresholds for the raw spectra of pea samples for starch **(A)**, crude protein **(B)**, moisture **(C)**, crude fiber **(D)**, and crude fat **(E)** (an outlier is an outlier when it exceeds half or more of the Mahalanobis distance of 1).

Between different pea varieties, there are certain differences between their physicochemical and nutritional qualities, which leads to certain differences between pea ingredients. The NIR is a very sensitive measuring instrument, and when this difference is large, then the detection threshold of the instrument is reached, resulting in an error large enough to affect the modeling results. The use of PCA combined with the Mahalanobis distance method to screen the outliers in the sample set can determine the outliers and reject the abnormal samples, thus improving the modeling effect of the infrared spectra of peas. As shown in Figure 5, the Mahalanobis distance distribution of the near-infrared raw spectra of the pea samples is plotted with the sample number as the horizontal coordinate and the Mahalanobis distance as the vertical coordinate. When the sample exceeds half or more of the value when the Mahalanobis distance is 1, this pea variety is considered to be an outlier, and then it needs to be rejected. However, if too many outliers were removed, then it may lead to the accuracy of the final NIR prediction model, so it was finally decided to remove 10 outliers and use the remaining 80 sets of spectral data for the construction of the NIR prediction model of pea’s basic ingredient content.

Compared with conventional methods, NIR detection technology has the advantages of non-destructiveness, rapidity, easy online monitoring and process control, wide applicability, and economic benefits, with specific parameter comparisons as shown in Table 5 (40).

**Table 5:** Comparison of metrics, such as accuracy, with those documented in analogous literature.

The sample size for the experiment after removing outliers	*n* = 80
Chemical method	Starch: GB 5009.9–2016 (27).
	Crude protein: GB 5009.5–2016 (28).
	Moisture: GB/T 21305–2007 (29).
	Crude fiber: GB/T 5009.10–2003 (31).
	Crude fat: GB 5009.6–2016 (30).
Near-infrared analytical methods	Starch model: the validation error is 0.21 g/100 g (71); the validation error is 7.05 g/100 g (34).
	Crude protein model: the validation error is 1.16 g/100 g (71); the validation error is 0.88 g/100 g (34); the validation error is 0.43 g/100 g (72).
	Moisture modeling: the validation error is 2.17 g/100 g (71).
	Crude fiber model: the validation error is 0.94 g/100 g (72); the validation error is 0.13 g/100 g (72).
	Crude fat model: the validation error is 0.45 g/100 g (72); the validation error is 1.12 g/100 g (73).

### 3.6 Construction of model

#### 3.6.1 Determination of spectral pretreatment method

To extract information related to chemical composition from spectra and eliminate interfering factors, it is crucial to use appropriate spectral preprocessing methods to build a stable and reliable model. Therefore, several spectral preprocessing methods were investigated to improve the signal-to-noise effect, including single and combined preprocessing methods. These methods were evaluated based on correlation coefficients and standard errors to determine the best pre-processing method. The effects of different spectral pre-treatments on the content of essential components of peas are shown in Supplementary Tables S2–S6. The optimal preprocessing method for the crude protein model was Derivative (2nd), for the crude fiber model was Derivative (1st) + Derivative (2nd), and for the crude fat model was Derivative (1st) + Derivative (2nd) + Detrend. The best preprocessing method for the starch model is Derivative (2nd), and the best preprocessing method for the moisture model is Derivative (2nd).

#### 3.6.2 Construction and verification of the model

To better and more intuitively evaluate the model’s generalization ability, this study utilized the optimal preprocessing method identified in section 3.6.1 to construct the model for pea basic component content. Specifically, RMSEC, RMSEP, *R*_c_^2^, and *R*_p_^2^ were used as the evaluation indexes of the model, in which the larger the values of *R*_p_^2^ and *R*_c_^2^, and the smaller the values of RMSEP and RMSEC, indicated the higher the model performance (18). The PLSR method was used to quantitatively analyze the data, aiming to identify the optimal regression curve and establish a quantitative analysis model for the basic components of peas.

To validate the accuracy of the pea basic ingredient content model, pea samples (*n* = 80) were validated using the Kennard-stone algorithm in which all pea samples (*n* = 80) were evenly divided into the calibration set samples and validation set samples in a ratio of 3:1, where 54 samples were used to construct the calibration model and 26 samples were used as validation set samples to verify the calibration model (48). The results are shown in Table 4. The pea starch *R*_C_^2^ was 0.99, RMSEC was 0.47 (Figure 4A); the pea crude protein *R*_C_^2^ was 0.98, RMSEC was 0.21 (Figure 4B); the pea moisture *R*_C_^2^ was 0.85, RMSEC was 0.90 (Figure 4C); the pea crude fiber *R*_C_^2^ was 0.93 and RMSEC was 0.25 (Figure 4D); the pea crude fat *R*_C_^2^ was 0.89 and RMSEC was 0.09 (Figure 4E); It was verified that the starch *R*_P_^2^ reached 0.77, with RMSEP was 0.86 (Figure 4A); the crude protein *R*_P_^2^ reached 0.77, with RMSEP was 0.24 (Figure 4B); the moisture *R*_P_^2^ was 0.71, with RMSEP was 1.00 (Figure 4C); the crude fiber *R*_P_^2^ was 0.72 and RMSEP was 0.59 (Figure 4D); the crude fat *R*_P_^2^ reached 0.88 and RMSEP was 0.25 (Figure 4E).

**Figure 6:**
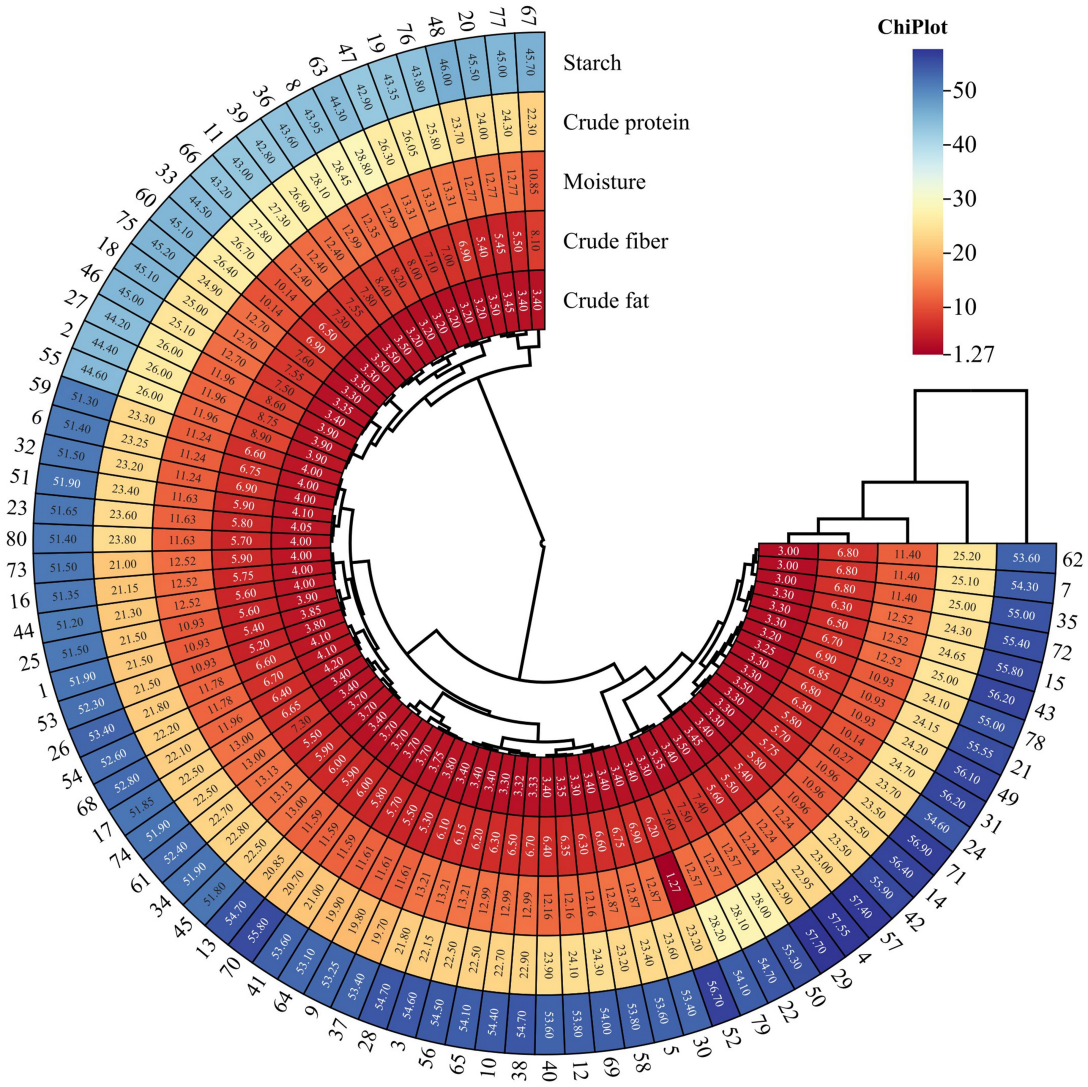
Heat map of correlation clustering depicting important component quantities in 80 pea varieties employed for modeling purposes.

As shown in Figure 6, the pea varieties (*n* = 80) used for modeling were classified into three categories. This demonstrates that the high-throughput NIR technique can quickly hierarchically classify different pea varieties compared to traditional methods. Circle heatmaps are plotted using ChiPlot (see Footnote 2).

A new method using a combination of portable rapid quality testing and near-infrared spectroscopy has been developed for the fast and non-destructive detection of the basic components in peas, to understand the correlation between the quality of pea varieties quickly and thus to carry out the work of pea variety quality grading. The technique starts with a routine chemical analysis of the sensory quality and basic components of peas, followed by correlation and cluster analysis of the data, and finally modeling and validation using near-infrared spectroscopy data.

Correlation analysis results showed that pea starch exhibited a substantial negative correlation with moisture, crude fiber, and crude protein while demonstrating a highly significant positive correlation with the thickness of the pea seed thickness; additionally, pea protein showed a significant positive correlation with crude fiber and crude fat. The combined contribution of PC1 and PC2 in the PCA was 51.2%. Cluster analysis showed that the different pea varieties used in the experiment could be classified into three groups. Starch, crude protein, and fat were classified into three classes, and specialized processing pea varieties were selected. Subsequent modeling using PLSR identified optimal preprocessing methods for various constituents: for pea starch, the Derivative (2nd) method resulted in a model *R*_C_^2^ was 0.99 and RMSEP was 0.86; for crude protein, the Derivative (2nd) method yielded *R*_C_^2^ was 0.98 and RMSEP was 0.24; for moisture, the Derivative (2nd) method *R*_C_^2^ was 0.85 and RMSEP was 1.00; for crude fiber, a combination of first and second derivatives provided *R*_C_^2^ was 0.93 and RMSEP was 0.59; and for crude fat, a combination of first and second derivatives plus detrending resulted in *R*_C_^2^ was 0.89 and RMSEP was 0.25.

This study has successfully demonstrated the use of near-infrared spectroscopy for the simultaneous, rapid, and non-destructive detection of essential ingredient content in whole peas. The technique significantly reduces the reliance on extensive laboratory equipment and lowers costs compared to traditional methods. It eliminates the need for sample preparation, and it takes only 5 s to obtain comprehensive data on the basic ingredient content of peas, which saves a great deal of experimental time. This will allow a quicker understanding of the relevance of the quality and grading of the different varieties of peas, which will lead to the selection of specialized varieties for pea processing. The high efficiency, multifunctionality, and broad applicability of NIR detection technology offer substantial advantages, facilitating the work of breeding experts in developing specialized varieties and assisting processing enterprises in establishing dedicated raw material bases. At the same time, near-infrared spectroscopy technology can not only quickly detect the basic composition content of peas, but also realize the rapid, non-destructive detection of soybeans, lentils, chickpeas, chickpeas and other leguminous crops, and analyze the proteins, lipids, moisture, fibers and other nutrients in them, to provide a scientific basis for the quality assessment of leguminous crops. In addition, near-infrared spectroscopy can be used in breeding, harvesting and sorting to realize the monitoring of the dynamic trend of the internal composition of the fruit, as well as the determination of the optimal harvesting period, thus improving the superiority rate of the fruit and market competitiveness. In the future, NIR spectroscopy technology is expected to be applied in more agricultural product testing fields, such as agricultural product quality and safety testing, origin traceability and crop breeding. Meanwhile, combined with advanced technologies such as IoT and AI, NIR spectroscopy technology will further expand its application scenarios and advantages, injecting new vitality into the development of modern agriculture.

## Data Availability

The datasets presented in this study can be found in online repositories. The names of the repository/repositories and accession number(s) can be found in the article/Supplementary material.

## Footnotes

^1^ https://www.figdraw.com/#/

^2^ https://www.chiplot.online/gene_cluster.html
