# Supplementary material for: High-throughput near-infrared spectroscopy for detection of major components and quality grading of peas
Source: Front Nutr. 2024 Dec 9;11:1505407. doi: 10.3389/fnut.2024.1505407 (PMC11663664; doi:10.3389/fnut.2024.1505407)
Supplement: Supplementary file 1 [file Data_Sheet_1.PDF]

Supplementary Material

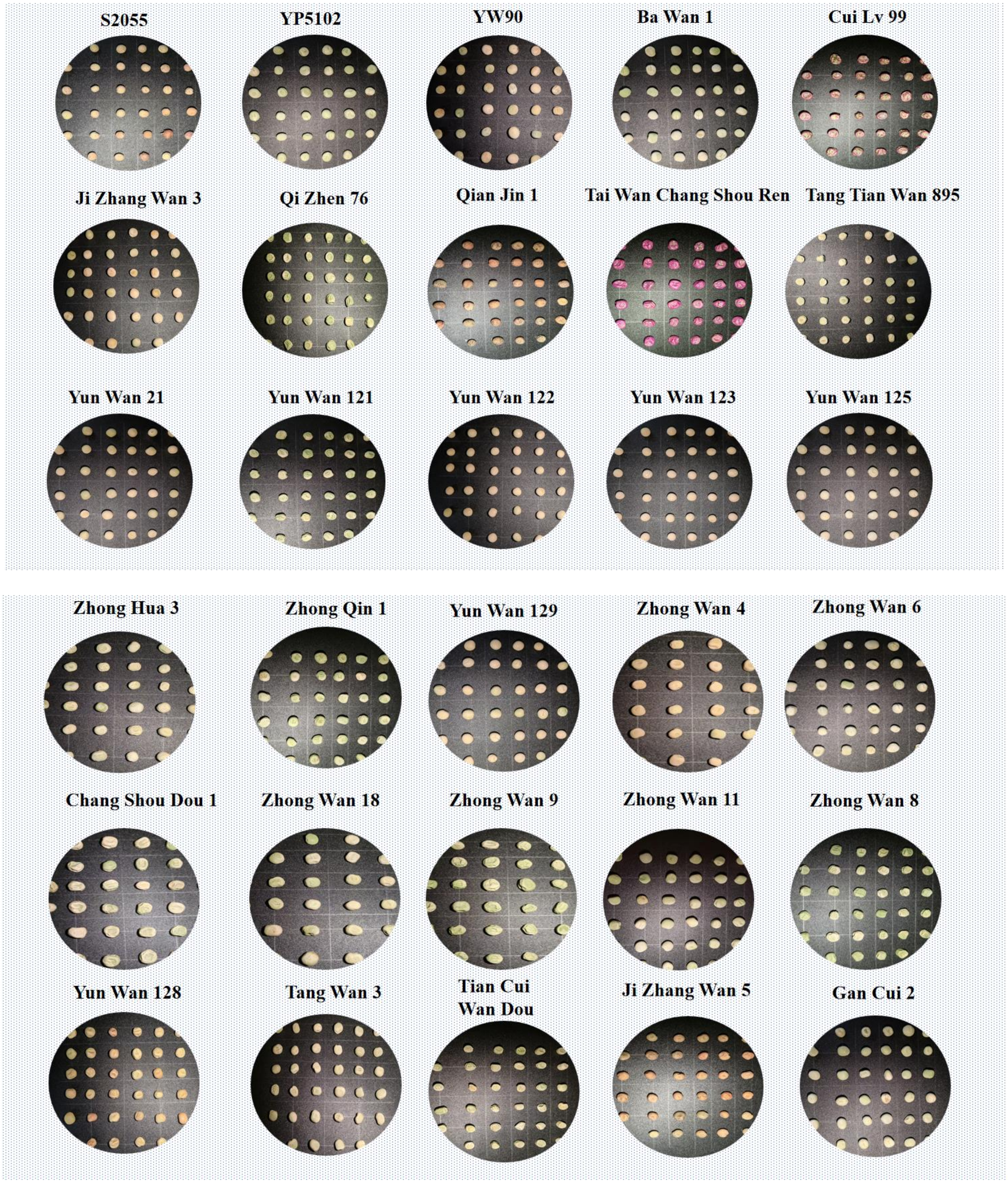

Supplementary Figure S1. Different varieties of pea seeds.

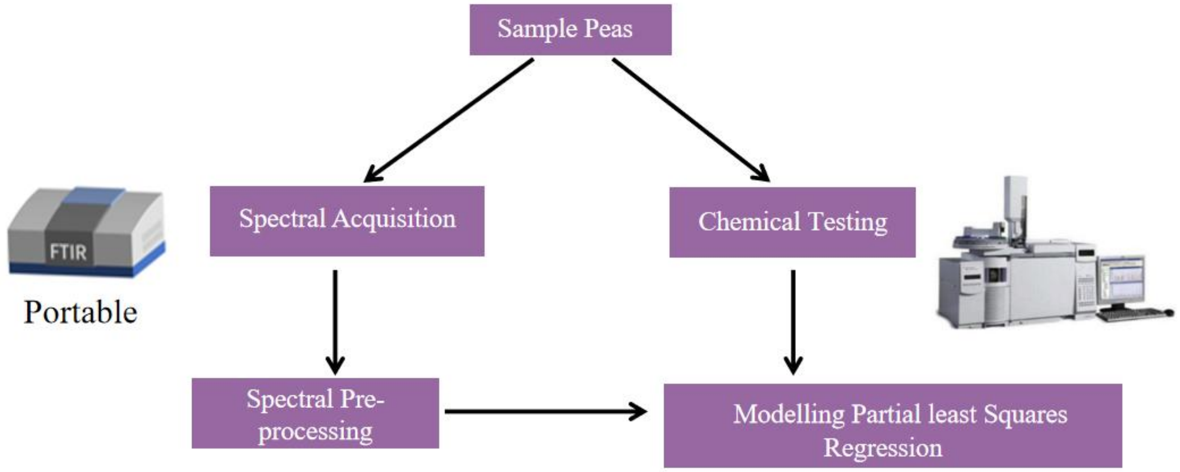

Supplementary Figure S2. Workflow of the study.

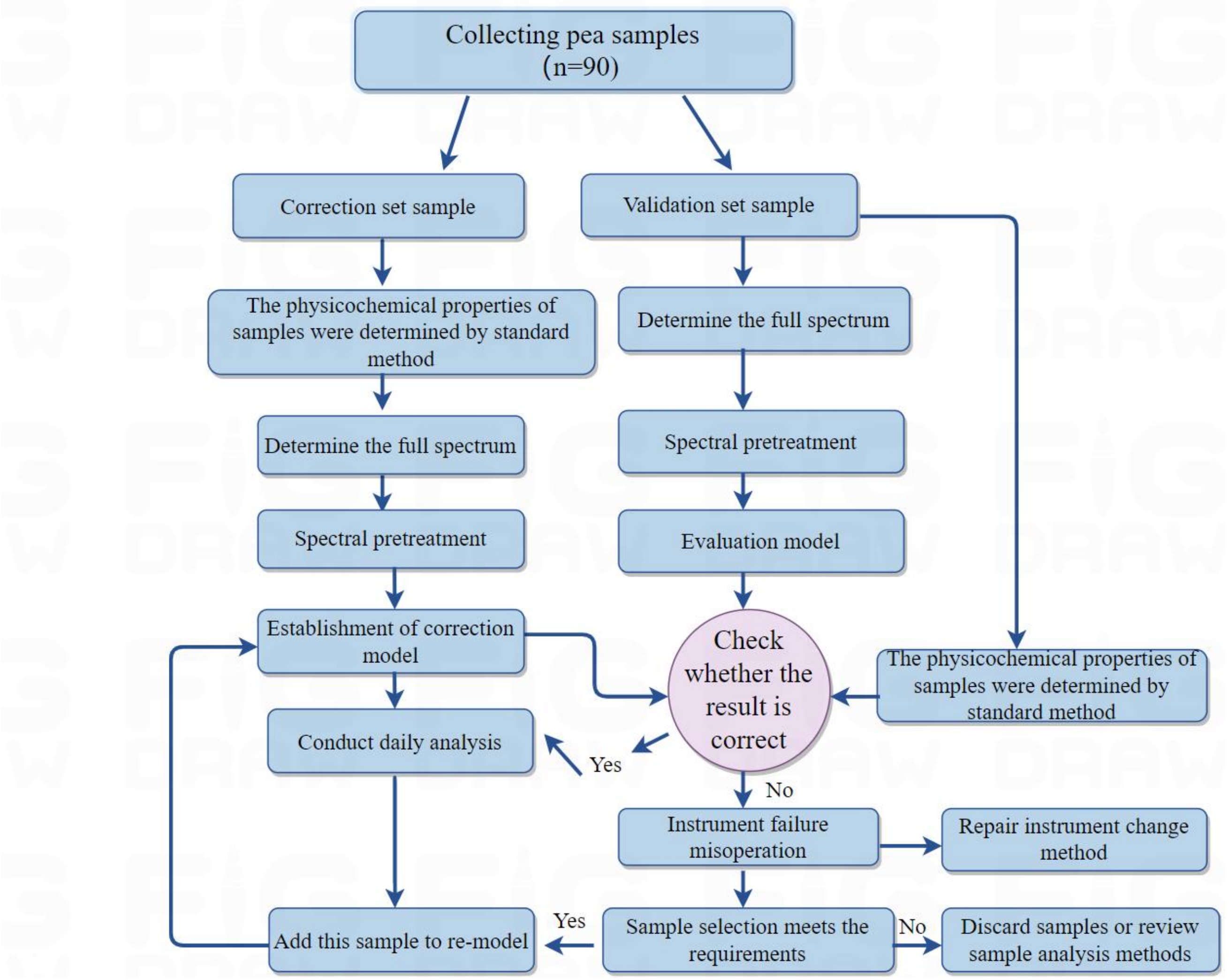

Supplementary Figure S3. Analysis flow chart of near-infrared spectroscopy.

Supplementary Table S1. Performance parameters of tachymeter

Performance index	Specific parameter
Light source lifetime/h	>40000
Wavelength accuracy /nm	2
Spectral sampling interval /nm	6
Spectral noise	0.0001
Detector	128 pixels uncooled indium gallium arsenic
Scanning time /s	2.4
Scan times	5
Integration time /ms	12.7

Supplementary Table S2. Effects of different spectral pretreatment on PLSR model of pea starch

Pretreatment method	Starch (n=80)			
	R_C	SEC	R_{CV}	SECV
Raw	0.40	2.47	0.21	3.07
MSC	0.55	2.02	0.30	2.86
SNV	0.55	2.02	0.35	2.67
Detrend	0.63	1.74	0.48	3.27
Baseline	0.50	2.19	0.27	3.91
Normalization	0.50	2.17	0.31	3.79
Derivative(2nd)	0.93	0.21	0.79	1.05
Derivative(1st)	0.82	1.91	0.64	2.70
Savit-Golay + Derivative(1st)	0.50	2.19	0.26	4.00
Savit-Golay + smoothing	0.15	3.15	0.09	4.41
Derivative(1st) + Derivative(2nd)	0.90	0.41	0.73	1.44
Derivative(1st) + Derivative(2nd) + SNV	0.88	1.55	0.72	1.31
Derivative(1st) + Derivative(2nd) + SNV+MSC	0.90	1.50	0.75	1.24

Supplementary Table S3. Effects of different spectral pretreatment on PLSR model of pea crude protein

Pretreatment method	Crude protein (n=80)			
	R_C	SEC	R_{CV}	SECV
Raw	0.24	1.78	0.13	1.93
MSC	0.33	1.67	0.15	1.93
SNV	0.53	1.40	0.31	1.74
Detrend	0.69	1.18	0.45	1.54
Baseline	0.43	1.55	0.24	1.81
Normalization	0.45	1.52	0.21	1.86
Derivative(2nd)	0.85	0.79	0.43	1.61
Derivative(1st)	0.20	1.82	0.13	1.91
Savit-Golay + Derivative(1st)	0.56	1.36	0.30	1.75

Pretreatment method	Crude protein (n=80)			
	R_C	SEC	R_{CV}	SECV
Savit-Golay + smoothing	0.23	1.79	0.11	1.95
Savit-Golay + Derivative(1st) + Derivative(2nd)	0.81	0.88	0.37	1.71
Savit-Golay + Derivative(1st) + Derivative(2nd) + Detrend	0.58	1.33	0.30	1.77

Supplementary Table S4. Effects of different spectral pretreatment on pea water PLSR model

Pretreatment method	Moisture (n=80)			
	R_C	SEC	R_{CV}	SECV
Raw	0.03	1.47	0.01	1.50
MSC	0.01	1.49	NA	1.53
SNV	0.01	1.49	NA	1.56
Detrend	0.06	1.45	0.01	1.50
Baseline	0.05	1.45	0.00	1.52
Normalization	0.07	1.44	0.02	1.49
Derivative(2nd)	1.00	0.00	1.00	0.00
Derivative(1st)	0.03	1.47	0.01	1.51
Savit-Golay + Derivative(1st)	0.08	1.43	0.01	1.52
Savit-Golay + smoothing	0.03	1.47	0.00	1.51
Savit-Golay + Derivative(1st) + Derivative(2nd)	0.02	1.48	0.00	1.53
Savit-Golay + Derivative(1st) + Derivative(2nd) + Normalization	0.03	1.48	0.01	1.50

Supplementary Table S5. Effects of different spectral pretreatment on PLSR model of pea crude fiber

Pretreatment method	Crude fiber (n=80)			
	R_C	SEC	R_{CV}	SECV
Raw	0.25	0.77	0.13	0.83
MSC	0.46	0.65	0.176	0.84
SNV	0.46	0.65	0.19	0.82
Detrend	0.27	0.76	0.20	0.80
Baseline	0.43	0.67	0.21	0.81
Normalization	0.17	0.81	0.07	0.89
Derivative(2nd)	0.81	0.38	0.54	0.61
Derivative(1st)	0.67	0.51	0.36	0.72
Savit-Golay + Derivative(1st)	0.43	0.67	0.18	0.83
Savit-Golay + smoothing	0.26	0.76	0.14	0.83
Derivative(1st) + Derivative(2nd)	0.83	0.37	0.54	0.61
Derivative(1st) + Derivative(2nd) + SNV	0.82	0.37	0.36	0.73

Pretreatment method	Crude fiber (n=80)			
	R_C	SEC	R_{CV}	SECV
Derivative(1st) + Derivative(2nd) + SNV + MSC	0.82	0.37	0.58	0.58

Supplementary Table S6. Effects of different spectral pretreatment on PLSR model of pea crude fat

Pretreatment method	Crude fat (n=80)			
	R_C	SEC	R_{CV}	SECV
Raw	0.18	0.27	0.09	0.29
MSC	0.15	0.27	0.13	0.28
SNV	0.15	0.27	0.11	0.28
Detrend	0.39	0.23	0.18	0.27
Baseline	0.17	0.27	0.12	0.28
Normalization	0.17	0.27	0.11	0.28
Derivative(2nd)	0.81	0.13	0.59	0.19
Derivative(1st)	0.80	0.13	0.61	0.19
Savit-Golay + Derivative(1st)	0.15	0.27	0.10	0.28
Savit-Golay + smoothing	0.18	0.27	0.09	0.28
Derivative(1st) + Derivative(2nd)	0.85	0.11	0.69	0.16
Derivative(1st) + Derivative(2nd) + Detrend	0.85	0.11	0.70	0.16
Derivative(1st) + Derivative(2nd) + Detrend + Normalization	0.37	0.23	0.21	0.27
